# Anticancer Compounds Derived from Marine Diatoms

**DOI:** 10.3390/md18070356

**Published:** 2020-07-09

**Authors:** Hanaa Ali Hussein, Mohd Azmuddin Abdullah

**Affiliations:** 1 Institute of Marine Biotechnology, Universiti Malaysia Terengganu, Kuala Nerus 21030, Terengganu, Malaysia; hanaazahraa85@gmail.com; 2 College of Dentistry, University of Basrah, Basrah 00964, Iraq

**Keywords:** diatoms, bioactive molecules, anticancer activity, nanomedicine, drug delivery system, biosilica

## Abstract

Cancer is the main cause of death worldwide, so the discovery of new and effective therapeutic agents must be urgently addressed. Diatoms are rich in minerals and secondary metabolites such as saturated and unsaturated fatty acids, esters, acyl lipids, sterols, proteins, and flavonoids. These bioactive compounds have been reported as potent anti-cancer, anti-oxidant and anti-bacterial agents. Diatoms are unicellular photosynthetic organisms, which are important in the biogeochemical circulation of silica, nitrogen, and carbon, attributable to their short growth-cycle and high yield. The biosilica of diatoms is potentially effective as a carrier for targeted drug delivery in cancer therapy due to its high surface area, nano-porosity, bio-compatibility, and bio-degradability. In vivo studies have shown no significant symptoms of tissue damage in animal models, suggesting the suitability of a diatoms-based system as a safe nanocarrier in nano-medicine applications. This review presents an overview of diatoms’ microalgae possessing anti-cancer activities and the potential role of the diatoms and biosilica in the delivery of anticancer drugs. Diatoms-based antibodies and vitamin B12 as drug carriers are also elaborated.

## 1. Introduction

Cancer is the major cause of morbidity and mortality worldwide, with an estimated 18.1 million new cases and 9.6 million deaths in 2018 [1]. There are over 200 types of cancers, and some can spread to other tissues in the body, leading to metastases and death [2]. Cancer progression is caused by the damaged DNA, abnormal DNA repair mechanism, activation of cancerous tumors, damaged tumor suppression activity, and promotion of cellular survival by angiogenesis, and metastasis. Cancer incidences have been projected to increase globally by about 68% in 2030. This calls for concerted efforts to discover novel chemotherapy, especially for prevalent cases such as lung/bronchial cancer in males, and breast cancer in females [3]. Chemotherapies are the first-line of treatment that could destroy or prevent the growth of cancer cells. There are, however, side-effects associated with chemotherapeutic drugs, which may lead to baldness or anorexia, and most of the drugs have some degree of harmfulness ranging from mild reactions to severe life-threatening effects [4]. Anticancer agents can inhibit the activity of oncogene by up or down-regulating successive signals of oncogene activation, or by activating the production of antitoxins, or the inhibitors of histone-deacetylases (HDAC), topoisomerase, acetyl-histones acetyltransferases (HATs), cyclin-dependent kinase, and DNA methyl-transferases [5]. 

**Figure 1:**
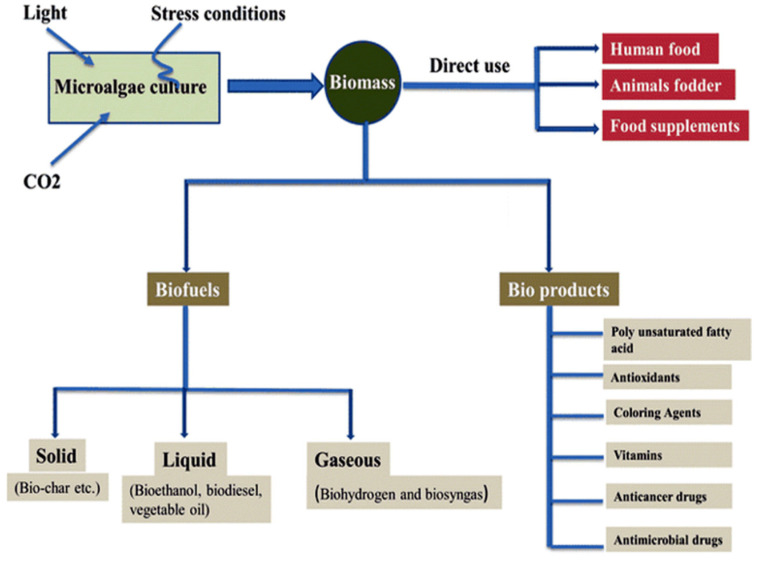
The microalgal biomass as a promising source for biofuel and bioproducts (Reprinted from [17], with permission from Springer Nature).

The discovery of cancer therapies from marine sources is highly relevant to combat cancer. More than 60% of anti-tumor drugs are derived from natural sources, including the confirmed pharmaceuticals and molecules, which are currently under clinical trials [6]. Biologically-active molecules isolated from nature have greatly contributed towards the development of new drugs for different human diseases [7]. Some of the anti-oxidants could prevent cells from DNA damage, or induce the repair mechanisms of abnormal DNA. Marine-derived microorganisms produce a large number of bioactive metabolites, with very unique structures, due to their special habitats, culture conditions, and isolation methods. This has led to the discovery of new classes of chemicals with therapeutic activities. Numerous studies have reported that these metabolites exhibit different biological activities such as anticancer, antimicrobial, antiviral, and anti-inflammatory activities [8]. Marine microalgae are potential sources of biochemicals for diverse applications in biofuel industries, food, pharmaceutical, nutraceutical, and cosmetics, as shown in Figure 1. They are rich in lipids, omega-3 fatty acids, carotenoids, pigments, vitamins, and polysaccharides, which confer different biological activities. There has been increasing interest in their uses as functional foods, nutrients, and supplements [2]. A large number of microalgal species are still largely unexplored and untapped for the identification of novel compounds [4]. Microalgae have shorter generation times than terrestrial plants, are easy to culture, and allow for a more eco-friendly approach in drug discovery development, without excessive sacrifice of marine resources, or large scale devastation from sampling and collection of specimens [9]. 

**Figure 2:**
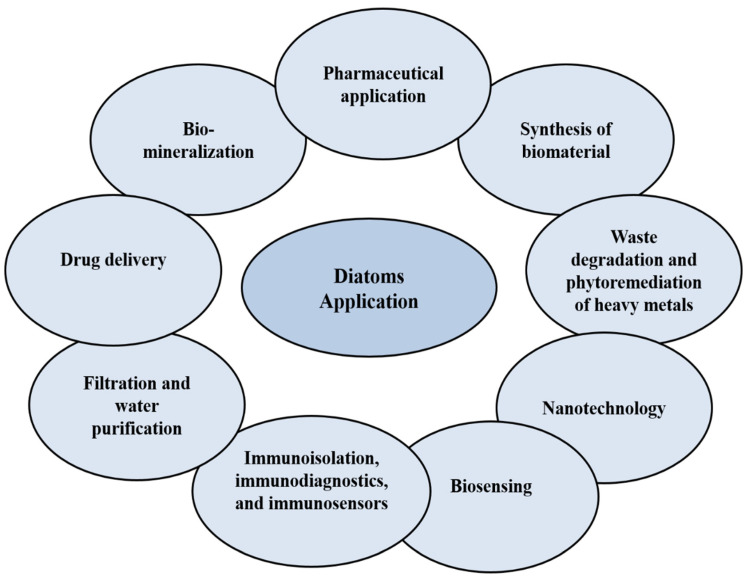
Applications of diatoms in different fields.

Diatoms are single-cell eukaryotic microalgae that act as photosynthetic organisms in the oceans, or in natural environments where water is present. The wide distribution, abundance and the diversity of chemical compounds make them ideal to be explored for applications such as anticancer [10,11] and antioxidative agents [12], for healthy foods and sources of drugs, in the synthesis of biomaterials and nanotechnology (drug delivery, molecular separation, biomimetic, photonic, structural materials, electronic, optical, and biosensing) [13,14,15,16], for the development of fuel synthesis, in the bioremediation of waste, heavy metals and polluted water [14,15], and for biomineralization [16] (Figure 2). From an ecological perspective, diatoms can produce 40% of the organic carbon from the ocean’s annual production, and 20% of the oxygen that living beings breathe [13]. Generally, diatoms are classified into two types—centric diatoms, which show radiate symmetry, and pennate diatoms, which have binary symmetry [13]. In this review, the potential of anticancer compounds derived from marine diatoms’ microalgae are discussed. The future of diatoms as a source of bioactive compounds, or as a drug delivery nanocarrier, is also elaborated.

## 2. Characteristics

Diatoms are unicellular, eukaryotic organisms that inhabit marine, freshwater and terrestrial environments [18]. They can play a global role in the productivity and biogeochemical circulation of silica, nitrogen, and carbon, principally because of their short growth-cycle and high yield [17]. Physiologically, diatoms may lead to the discovery of new pathways and novel compounds, and also assist in understanding the evolutionary history of eukaryotes and their environmental adaptation [15]. Diatoms appear to contain similar structures to the eukaryotes, in having the nucleus, secretory apparatus, and mitochondria. However, their plastidial ultrastructure is actually more complex. The stroma (plastid-plasma) is separated from the cytoplasm through four membranes. The red or green algae and plants are separated by two membranes [14]. Most diatoms are identified by the specific shape of the amorphous silica cell-walls, which are different between species, based on the nanometer sizes [19]. The nano-micrometer-sized pores and silica-nanospheres can provide large surface area for the diatoms [20].

**Figure 3:**
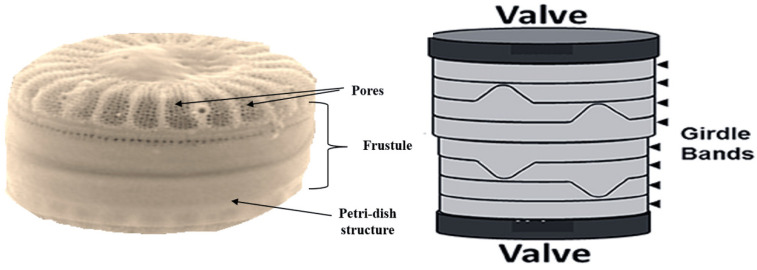
Image of the diatoms’ cell wall (frustule) showing that they contain two thecae over-lapped structures, similar to a petri-dish, each half consisting of a valve and different girdle-bands, extending around the circumference of the cell, with nanopores at different scales (Modified from [13]).

Diatoms are rich not only in minerals, but also in the primary and secondary metabolites such as the saturated and unsaturated fatty acids, esters, acyl lipids, sterols, proteins, and flavonoids. These compounds have been reported to exhibit anti-cancer, anti-oxidant, and anti-bacterial activities [21]. The monogalactosyl diacylglycerol extracted from the marine diatom *Phaeodactylum tricornutum* has shown anticancer activity through the induction of apoptosis. The high phenolic contents may contribute towards the free radical scavenging activities observed [21]. As shown in Figure 3, the diatom cell wall (frustule) contains two thecae over-lapped structures, akin to a petri-dish, each half consisting of a valve and different girdle-bands, extending around the circumference of the cell [19]. The frustule consists of silica and organic materials such as carbohydrates and glycoproteins. The decomposition of the diatom frustules has resulted in the sea bottom being covered with sediments called diatomite or diatomaceous earth (DE). These highly porous materials are suitable to be developed as adsorbents, mineral filters, abrasives, anticaking factors, or for materials isolation [21]. The frustules of diatoms can be used as complex molds in the decoration of biomolecules at both the micro and/or nano-scale, taking advantage of their complex structural engineering [16]. The diatomaceous filters have found several applications such as in fluid filtration, DNA purification, and adsorption of heavy metals [14]. In nanotechnology, the complex nanoscale structure of the silica shell is beyond the existing human engineering capabilities. The formation of bio-glass diatoms is achieved in moderate physiological conditions, without any need for high pressure, high temperatures, or the use of acidic or toxic chemicals. Other organisms such as some plants, chrysophytes, and desmosponges have similar capabilities of manufacturing the silica-based structures, but diatoms are shown to be at the top in the world of silica-cycles [19]. The metabolic pathways associated with silica cell wall formation are largely unknown. This could open up possibilities of discovering new proteins and enzymes that may be commercially useful [22].

The frustule morphology and size may change with vegetative cell division. For instance, *Skeletonema marinoi* forms broad chains, with full silicate frustules [23]. *Phaeodactylum tricornutum* shows many shapes such as oval, triradiate and fusiform, and can change from one shape to another over time. When grown in cultures, the triradiate and fusiform can produce long chains where the frustules are completely organic. In the oval type, the valves may be organic, or one of the valves may have a small silica frustule covered by an organic wall [24]. *Pseudo-nitzschia australis* cells are characterized by narrow and spindle shapes, with apically asymmetric valves combined in stepwise chains, with interlaced valve ends. The valve face is surrounded by slits and pores (striae, fibulae, interstriae). The raphe is outside of the center and not raised above the valve [25].

## 3. Anticancer Compounds from Diatoms

The identification of cytotoxic metabolites for many years has led to the advancement in anti-cancer treatments. However, progress in cancer therapy has been hampered by the inability to identify the unique biochemical features of malignant tumors, which can be utilized to selectively target the cancer cells [26]. The detection and evolution of anti-cancer drugs (with cytotoxic factors), is very different from the drug development for many other diseases. Cytotoxic agents with highly toxic effects on the cancer cells could have also affected the healthy normal cells [27]. Microalgae, in general, have received increasing interest as the extracts have exhibited anti-cancer (anti-proliferative) activities in several types of cancer [2,27]. However, despite increasing efforts, only a number of these specific metabolites with anticancer activities show potential to be developed further as anticancer drugs. A study to discover new diatoms as potential sources of anticancer compounds has examined 21 diatoms, 4 flagellates, and 7 dinoflagellates grown in various cultivation conditions [9,28]. Only one extract (FE60), from two different FE60 *Skeletonema marinoi* clones, has demonstrated cytotoxicity against human A2058 melanoma cells, while the other clone (FE6) does not show any activity at any growth conditions. These results confirm the differences in secondary metabolism between the clones of the same species, and the effects of the different culture conditions on the production of biologically active molecules [9]. Different types of compounds such as Monoacylglycerides (MAGs), Oxylipins (OXLs), Chrysolaminaran polysaccharide, Fucoxanthin, Fatty alcohol ester (nonyl 8-acetoxy-6-methyloctanoate, NAMO), Adenosine and metabolites, Stigmasterol and Marennine, and Haslenes (hasla-6(17),9,13,23- tetraene) lipid, have been identified as the potential anticancer compounds from diatoms (Table 1). 

### 3.1. Monoacylglycerides (MAGs) 

**Figure 4:**
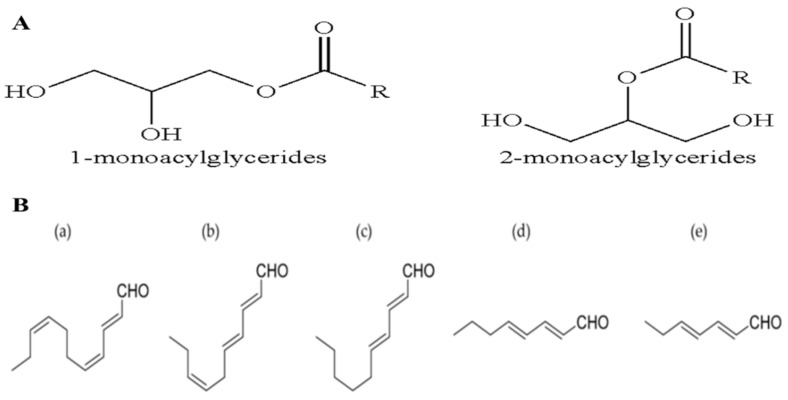
Chemical structure of: (**A**) monoacylglycerides; (**B**) polyunsaturated aldehydes: (a) 2-trans-4-cis-7-cis-decatrienal; (b) 2-trans-4-trans-7-cis-decatrienal; (c) 2-trans-4-trans-decadienal; (d) 2-trans-4-trans-octadienal; (e) 2-trans-4-trans-heptadienal (Modified from [2]).

MAGs have been isolated from *Skeletonema marinoi*, which exhibit potent anticancer effects on colon cancer (HCT-116) and haematological cancer (U-937) cell lines, with IC_50_ of 5 µg/mL after 24 h treatment and without affecting the normal MePR-2B cells. MAGs cause cell death by the induction of apoptosis in HCT-116 and U-937 cells, through caspase 3/7 [28]. The LC–MS analyses show that the MAGs mixture consists of unsaturated C20:5 (eicosapentaenoic acid, EPA), C16, and C22:6 (docosahexaenoic acid, DHA) fatty acids, with small amounts of C18 and C20:4 (arachidonic acid, EHA) fatty acids (Figure 4A) [28]. The MAG-ω3, which includes MAG-EPA and MAG-DHA, exhibits beneficial pharmacological and medicinal effects in several diseases including lung and colorectal adenocarcinoma [29]. The anticancer effects of MAG-EPA against HCT-116 cell lines show the IC_50_ of 1.32 ± 0.05 µM, and the in vivo study in the xenograft mouse model exhibits a decrease in the tumor growth to about 75% (38.4 ± 14.2 mm^3^) after 27 days [30]. The absorption ability of ω-3 PUFA-MAG is observed to be the best as compared to the intake rate of the relatively free PUFA, confirming MAGs as having major medicinal benefits in the therapy for, and protection from, inflammatory activity and tumor generation [29,30]. The plasma lipid analyzed suggests increased levels of DHA, EPA, and oxidizing metabolites in the MAG-EPA-supplemented animals, with increased ω3 (EPA) levels specifically in the lung, and lower levels of linoleic acid and arachidonic acid. This confirms that MAG-ω 3 is non-toxic and present in both blood circulation and specific organs [29].

### 3.2. Oxylipins (OXLs)

Marine diatoms can produce 30% of OXLs’ metabolites. The conversion of polyunsaturated fatty acids (PUFAs) into different types of OXLs is mediated by lipoxygenase/hydroproxidylase [31,32]. OXLs are produced from the incorporation of oxygen into the carbon chains of PUFAs, which also normally act as a chemical medium in many environmental and physiological processes in freshwater and marine diatoms [32]. In plants, the 18C fatty acids, such as α-linolenic and linoleic acids, act as important sources of OXLs, whereas microalgae may convert the 18C and/or 20C fatty acids. Normally, the green microalgae metabolize at the C-9 and C-13 sites of the 18C fatty acids, while the brown microalgae metabolize the 18C and 20C fatty acids by lipoxygenases with *n*-6 specificity [33]. The transformations of these fatty acids vary in marine algae, but are crucial for chemical protection, signaling, and responses to stresses. These are mediated by polyunsaturated aldehydes (PUAs), including decadienal (C10:2 *n*-3, DD), heptadienal (C7:2 *n*-3, HD), octatrienal (C8:3 *n*-1, OD), octadienal (C8:2 *n*-4, OD), C18:3, C22:6 (DHA), and C20:5 (EPA) [18,33,34,35] (Figure 4B). The PUAs are usually produced only when the diatom cells are damaged. The mass, structural diversity and the amount of PUAs vary greatly, based on the species and environmental conditions. Microalgal OXLs, such as the PUAs extracted from PUFAs, could inhibit the growth of predators, and stimulate many responses in the marine ecosystem such as the cell-to-cell signalling, allelopathic and anti-bacterial activities [11,31,32]. The PUAs (DD, HD, and OD) derived from *Skeletonema marinoi* have shown anticancer activity against lung cancer (A549) and colon cancer (COLO 205) cell lines, but with no effects on the normal epithelial (BEAS-2B) cell line after 48 h treatment [11]. The DD, 2-trans,4-cis,7-cis-decatrienal, and 2-trans,4- trans,7-cis-decatrienal have all shown anti-proliferative activities and apoptotic effects in the human adenocarcinoma (CaCo2) cell line [11]. The cell-lines treated with DD have been shown to induce extrinsic apoptotic pathways. Other anti-cancer drugs, on the other hand, induce intrinsic apoptotic pathways against A549 cells through the activation of tumor-necrosis factor receptor 1 (TNFR1) and the Fas-associated death domain (FADD), leading to cell death by caspase-3, without stimulating the survival path of the receptor-interacting protein (RIP) [11]. *Skeletonema costatum, Pseudo-nitzchia delicatissima,* and *Thalassiosira rotula* produce three anti-proliferative aldehydes, which induce low hatching values in copepods. Compounds, such as 2-trans-4-trans-7-cis-decatrienal, 2-trans-4-trans-decadienal, and 2-trans-4-cis-decatrienal (Figure 4B), have been suggested to be responsible for the reproductive failure in the copepods when the main food source is diatoms [33].

### 3.3. Chrysolaminaran Polysaccharide

**Figure 5:**
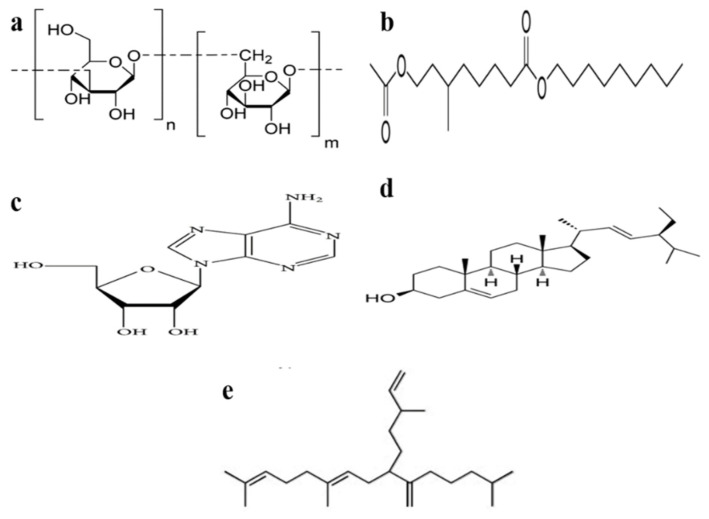
Chemical structure of: (**a**) Chrysolaminaran polysaccharide; (**b**) Nonyl 8-acetoxy-6-methyloctanoate (NAMO); (**c**) Adenosine; (**d**) Stigmasterol; (**e**) Hasla-6(17),9,13,23- tetraene. (Modified from [2]).

The organic mass of diatoms contains 10 to 80% polysaccharides, which play a role in their metabolism, in the structural make-up, storage, and extracellular components [18]. Chrysolaminaran polysaccharides (Figure 5a) have been used in cancer immunotherapy as immuno-modulators, and to reduce infections, and are therefore seen as an alternative cytotoxic drug [18]. Chrysolaminaran is produced by *Synedra acus* as a water-soluble biopolymer in storage compartments [2]. The water-soluble carbohydrates are more biologically accessible than the insoluble-starch, and are therefore useful for energy applications [2]. The anti-cancer activities of the chrysolaminaran have been exhibited on the HT-29 and DLD-1 colon cancer cells, with the IC_50_ of 54.5 µg/mL and 47.7 µg/mL, respectively, after 72 h. The activity against the normal human cells is, however, not reported [18], though it is the main drug for treating colon cancer in humans. Novel chrysolaminarin (CL2), isolated from the marine diatom *Odontella aurita* with a molecular weight of 7.75 kDa, has shown high antioxidant activity (83.54% ± 6.71% at 10 mg/mL), suggesting its potential as a natural anti-oxidant for pharmaceutical, aquaculture, and food applications [12]. 

### 3.4. Fucoxanthin

**Figure 6:**
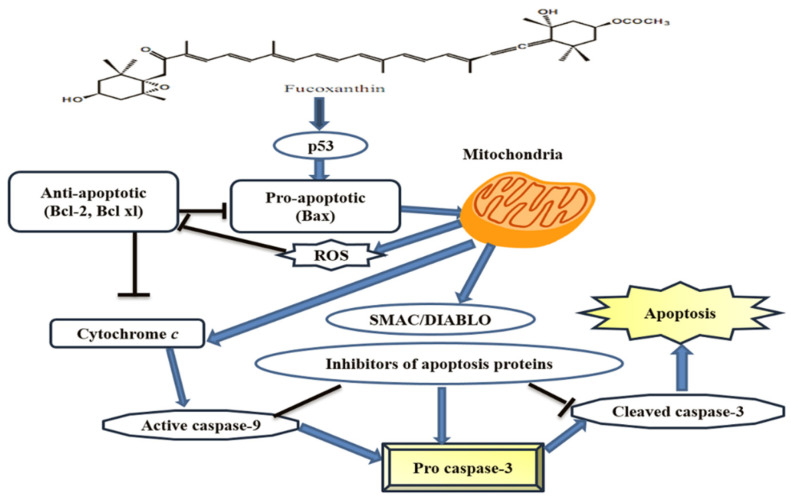
Chemical structure and the apoptotic signaling pathway of fucoxanthin (Modified from [49]).

Fucoxanthin is one of the most abundant carotenoids, forming more than 10% of the total carotenoids in the marine environment [43]. Besides β-carotene and chlorophyll *a* and *c*, fucoxanthin, which is characterized by its orange color, is present in Bacillariophyta diatoms including *Chaetoseros* sp., *Cylindrotheca closterium*, *P. tricornutum*, and *O. aurita*. It is also present in Chromophyta (Heterokontophyta or Ochrophyta), which includes the brown seaweed (Phaeophyceae) [44]. Fucoxanthin shows potential anti-cancer, anti-oxidant, anti-inflammatory, antidiabetic, anti-hypertensive, and antiobesity activities, and has been used as animal-feed additive in aquaculture and poultry [45]. The diatom biomass contains ten times higher levels of fucoxanthin than macroalgae. Unlike macroalgae, however, diatoms can be grown with high biomass yield in indoor and outdoor systems, and not only in one season, but throughout the year. Diatoms can also be grown in a closed photo-bioreactor such as in the flat-panel airlift and bubble-columns bioreactor [37]. Fucoxanthin exhibits interactive activity with anticancer drugs and may be applicable in different types of cancer [46]. Fucoxanthin isolated from the dichloromethane fraction of *Chaetoceros calcitrans* has shown strong antioxidant activity, attributable to the presence of allenic bonds in the C-7 site, in combination with the carbonyl 5,6-monoepoxide, and acetyl region, with a high potency to quench the free-radicals [47]. The anti-proliferative activities of fucoxanthin isolated from *O. aurita*, against the NSCLC-N6 cell-line (derived from a human non-small-cell bronchopulmonary carcinoma), have been reported with the IC_50_ of 10 µg/mL after 72 h treatment. Fucoxanthin induces apoptosis, resulting in DNA fragmentation, and morphological changes such as decreasing cell sizes, rounding up of cells, condensation of chromatin, nuclei damage, and apoptotic body formation in the NSCLC-N6 cells [48]. Fucoxanthin isolated from diatom *P. tricornutum* has shown inhibition of the HepG2 cells by up to 58%, with similarly higher inhibition of the Caco-2 and HeLa cells than the positive control in 5% DMSO [37]. As shown in Figure 6, the anti-proliferative effects of fucoxanthin may be due to the induction of apoptosis through the activation of caspase 3/7. The inhibitory effects on the HeLa cells increase 4.6-fold at the higher concentration of fucoxanthin. 

### 3.5. Fatty Alcohol Ester (Nonyl 8-Acetoxy-6-Methyloctanoate, NAMO)

Nonyl 8-acetoxy-6-methyloctanoate (NAMO) (C_20_H_38_O_4_) is a novel fatty alcohol ester derived from the marine diatom *P. tricornutum* Bohlin. The compound is detected in the Electron Spray Ionization (ESI) negative ion mode at 341.14 *m/z*, and the structure (Figure 5b) has been confirmed by the Heteronuclear Single Quantum Coherence (HSQC) spectral data [3]. NAMO shows anticancer activity against human promyelocytic leukemia (HL-60) and A549 cells, with the IC_50_ of 22.3 and 50 µg/mL, respectively, after 48 h, without affecting the mouse melanoma (B16F10) cell lines. The apoptosis induction in HL-60-7 cells after NAMO treatment has been shown with the condensation of DNA in a dose-dependent manner, cell cycle arrest at the sub G1 phase, and an increase in the expression of a pro-apoptotic protein (Bax), p53, caspase 3, and the suppression of anti-apoptotic protein (Bcl-x) [3].

### 3.6. Adenosine and the Metabolites

Diatoms normally contain high amounts of adenosine (Figure 5c). Adenosine transfers energy and signals in the cells, and shows a wide range of cellular protection or prevention of tissue damage. It is therefore suitable for the treatment of chronic heart failure, and for anticonvulsant and anti-inflammatory activity. Adenosine inhibits cancer cell growth by the activation of caspases through both the intrinsic and extrinsic apoptotic signaling pathways [50]. The *P. tricornutum* extracts, for example, exhibit anti-leukemic activity, attributed to the presence of adenosine (0.17 µg/mg dry weight) [51]. At 0.31 μg/mg DW level in the diatoms biomass, the 50% lethal concentration (LC_50_) value of adenosine in IPC-81WT leukemic cells is 7 μM. Adenosine deaminase-sensitive compounds have shown anti-leukemic activity with the ability to induce cell death in different types of cells [51]. Adenosine elevates reactive oxygen species (ROS) in the cancer cells, leading to a positive feedback complex, which affects the mitochondrial membrane and increases the cell death signals [50].

### 3.7. Stigmasterol

Stigmasterol is a naturally produced 6-6-6-5 monohydroxy phytosterol, and is structurally similar to cholesterol, with 1.73 nm in size. It is characterized by a double bond at the C5-6 region, and the presence of one polar hydroxy end, and a large non-polar lipophilic steroid structure with a semi-flat and rigid 6-6-6-5 structure and a branched-chain at C10 (Figure 5d). The structure is unique and attractive for the study of self-assembly properties in many liquids [38,52]. Stigmasterol exhibits anti-cancer and antioxidant activities, with the ability to lower cholesterol in the blood, and block cardiovascular disorders [2]. The stigmasterol isolated from *Navicula incerta* has shown cytotoxicity against HepG2 cells with the IC_50_ of 8.25 µg/mL after 24 h treatment. The DNA damage and higher apoptotic events suggest that stigmasterol induces apoptosis through the intrinsic pathway in the mitochondria [38].

### 3.8. Marennine

Marennine is a water-soluble blue-pigment isolated from the diatom *Haslea ostrearia*. A method to extract and purify marennine has been developed, but its chemical structure could not hitherto be resolved. It however shows growth-inhibitory, anti-oxidant, allelopathic, anti-bacterial, and antiviral activities [53]. New species of the blue pigment diatoms, such as *H. silbo* sp. inedit, *H. provincialis* sp. inedit, and *H. karadagensis*, produce several marennine-like pigments, having the same chemical-family and activities [53]. The *H. ostrearia* strain, especially, has big potential for commercial applications in the cosmetics, health food, and aquaculture industries [53]. The *H.* ostrearia aqueous extract has shown anti-proliferative activity against skin cancer (M96), lung cancer (NSCLC-N6), and kidney cancer (E39) at the IC_50_ of 30.2, 34.2, and 57.8 µg/mL, respectively, after 72 h treatment. The extract induces apoptosis by the cell cycle arrest in the G1/S phase. Significant reduction in the tumor growth in the in vivo study has been reported with total inhibition observed at 100 mg/kg intravenous (IV) administration. The anticancer activities have been attributed to the pigments detected in the *H. ostrearia* crude extracts [54].

### 3.9. Haslene (Hasla-6(17),9,13,23-Tetraene) Lipid

Haslene (hasla-6(17),9,13,23- tetraene) is an unsaturated C_25_ fatty acid isolated from *H. ostrearia*. It is a highly branched isoprenoid (HBI) hydrocarbon (Figure 5e), found in aquatic environments. *H. ostrearia* has been identified as the natural source for the sedimentary haslene, deposited in the 500 L tank cultivation, containing 198 mg of the nonpolar portion [55]. The most bioactive haslene is in the unsaturated part. Unsaturation in haslene can be increased by increasing the temperature during the diatom growth [39]. Haslenes exhibit cytotoxic activity in the NSCLC-N6 cell-line with the IC_50_ of 3.8 µg/mL, and cell cycle arrest is observed at the G1/S phase, an event that indicates apoptosis [39].

### 3.10. Diatom Extracts 

Different solvent extracts of diatoms have also exhibited potent anticancer activities with the induction of apoptosis. *Chaetoceros calcitrans* ethanol extracts (EEC) show anti-cancer activity against MCF-7 and MCF-10A cells at the IC_50_ of 3.00 ± 0.65 and 12.00 ± 0.59 µg/mL, respectively, after 24 h treatment. The activity of EEC against MCF-7 cells may be due to the stimulation of apoptosis without cell cycle arrest. While the untreated cells only show 8.62 ± 0.19% and 1.28 ± 0.02% of early and late apoptosis, respectively, the treated cells with EEC show much increased early and late apoptosis at 49.84 ± 0.47% and 12.63 ± 0.24%, respectively. The apoptotic events, in the sub G0/G1 phase in the treated MCF-7 cells, increase by 34 and 16 times after 48 and 72 h, respectively, as compared to only six and seven times in the normal MCF-10A cells [41]. 

The *C. calcitrans* ethyl acetate extract (CEA) has exhibited cytotoxic activity against MDA-MB-231 cancer cell line at the IC_50_ of 60 µg/mL. The stimulation of early apoptosis in MDA-MB-231 cells treated with the CEA extract at 30, 60 and 120 μg/mL is enhanced by 8.15 ± 0.08, 11.9 ± 0.24 and 18.9 ± 0.27%, while the viable cell population decreased by 5.3, 9.3 and 20.7%, respectively. The highest late apoptotic event (13.00 ± 0.83%) is only achieved after the treatment with 120 μg/mL of the CEA extract. The results suggest that the induction of early apoptosis in the MDA-MB-231 cells is higher than the late apoptosis and necrosis [42].

The *Cocconeis scutellum* diethyl ether extract, especially the fraction rich in EPA, can reduce the viability of BT20 cells and induce apoptotic activities (more than 89.2% at 1 μg/well), by arresting the cell cycle in the S and G2-M phase, and activating caspases 8 and 3. The standard EPA exhibits the same apoptotic activity in the BT20 cells, confirming that the *C. scutellum* diatoms can be a potential source of anticancer compounds [40].

## 4. Diatoms for Drug Delivery Systems

**Figure 7:**
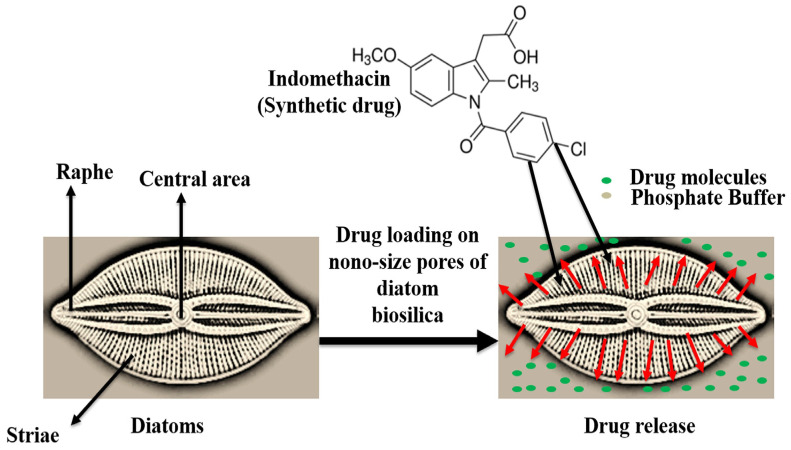
The mechanism of drug release from the porous diatom micro-shell (Modified from [58]).

Drug delivery systems (DDS) overcome the limitations of conventional pharmaceutical applications such as low solubility, low life-time circulation, systemic toxicity, and degradation [56]. The discovery of new drugs is also costly and time-consuming. The development of DDS and the use of nanotechnology in medicine has intensified in recent years with the aim to enhance the activity and effectiveness of present drugs, to reduce side effects, and also increase the life-span of patients [20,57]. The silica cell wall of diatoms (hydrated-silicon dioxide) or the frustule, can be harvested, isolated, and purified as a good source of cost-effective and eco-friendly silica [10]. New developments have been made on the silica shells of the diatoms as a promising biomaterial, taking advantage of the unique chemical, structural, optical, and mechanical properties that could provide a viable alternative to the normal or micro-particle DDSs [13]. Silica has a high surface area, with features such as nano-porosity, bio-compatibility, and bio-degradability, and the functionalized biosilica of diatom has been developed as a DDS [51]. As shown in Figure 7, the nano-sized, porous silica capsule can be exploited for drug carrier or release [52,53], where the drug molecules can be loaded on the internal and external surfaces of the diatoms [58].

**Figure 8:**
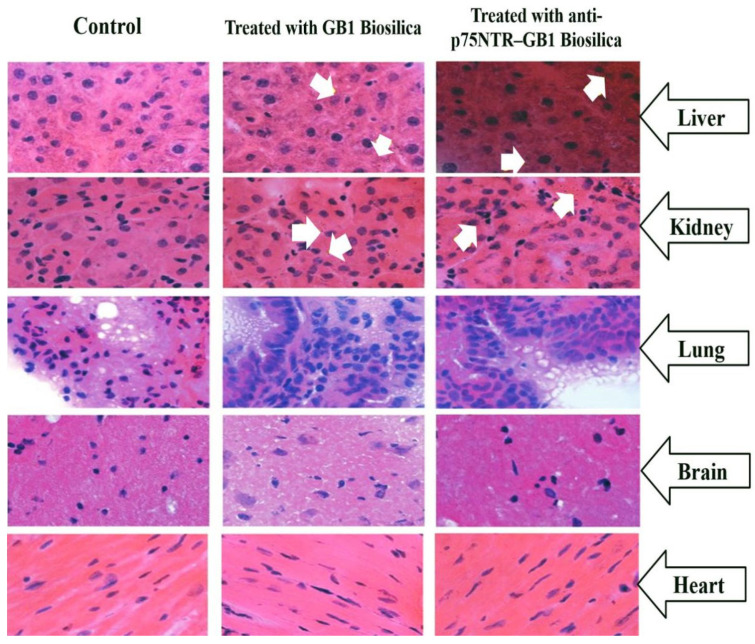
In vivo biological distribution and tissue damage studies upon treatment with GB1 and anti-p75NTR–GB1 diatoms biosilica. The tissues look normal. Some biosilica fractions appear in the kidney and liver samples of the treated mice, as shown by the white arrows (Modified from [61]).

An in vitro screening of diatom microfrustules for oral DDS has been tested against Caco-2/HT29 cells, using adenosine triphosphate (ATP) measurement to evaluate the cell viability. The diatoms’ microcapsules show low toxicity against Caco-2/HT29 cells at more than 1000 µg/mL dose after 24 h, suggesting their safe application [59]. The diatomite particles (DPs) of 300 nm size also exhibit no cytotoxicity against epidermoid carcinoma (H1355) cells, even at high concentrations of 300 µg/mL after 72 h treatment, further confirming the DPs as a safe carrier for drug delivery [60]. For in vivo study, the BLAB/c mice have been injected (intravenously) with the diatoms biosilica and monitored daily for eight days, and the main organs later harvested. The light microscopic studies on the tissue part (8 µm size) do not indicate any significant distortion of the main organs, including the brain, kidney, heart, lung, liver, and tail. However, the biosilica is detected in the kidney and liver, though not in the lung (Figure 8) [61]. The liver is expected to accumulate the silica as a result of particle absorption by the macrophage in the reticuloendothelial system [61]. Different strategies may be devised to modify the biophysical properties of biosilica, including the development of surface functionalization and composite materials, to improve drug loading, release efficiency, targeted delivery, and site-specific binding. [62]. However, despite the biocompatibility and purported safe use of diatomite biosilica, efforts must be made to understand the effects of overdoses and particle accumulation in key tissues and organs, especially in the animal model. 

### 4.1. Diatom-Based Nanoparticles (DNPs)

Nanoparticles (NPs) prepared in small sizes and high surface area could enhance the biological distribution of cancer drugs and increase the spreading time in the blood-stream. Drug-loaded NPs targeting only cancer cells must take cognizance of the selective pathophysiology of the tumor cells and should possess high permeability and retention effects in the specific tumor environment. Apart from passive targeting, active targeting of antibodies or ligands against tumors, to increase the selective therapeutics of the NPs, has been reported [56]. The preparation of diatomite nanoparticles (DNPs) and active biofunctionalized nanocarrier for delivery of anticancer drugs can be formulated as an alternative to the synthetic NPs [63]. The diatom-mediated gold-NPs have reportedly induced apoptosis induction and exhibited antimicrobial activity [21]. The anti-cancer drug delivery that uses three groups of surfactant-templated mesoporous silica NPs (Surf@MSNs) with a diameter of 150–660 nm, including Triton X-100 (Triton@MSNs, nonionic surfactant), sodium dodecylbenzene sulfate (SDBS@MSNs, anionic surfactant), and cetyltrimethylammonium bromide (CTAB@MSNs, cationic surfactant), have been reported [64]. The known anticancer drug CPT-11, templated into surfactant-free MSNs (CPT@MSNs), is used as a reference to determine the anticancer activity of the Surf@MSNs. The Surf@MSNs have shown the cytotoxic properties in the MCF-7 cells in the order of: CTAB@MSNs > SDBS@MSNs > Triton@MSNs. The CTAB@MSNs, prepared using mesoporous silica NPs at very low concentrations (2-15 mg/mL), have therefore successfully shown higher anticancer activity than the CPT@MSNs [64].

**Figure 9:**
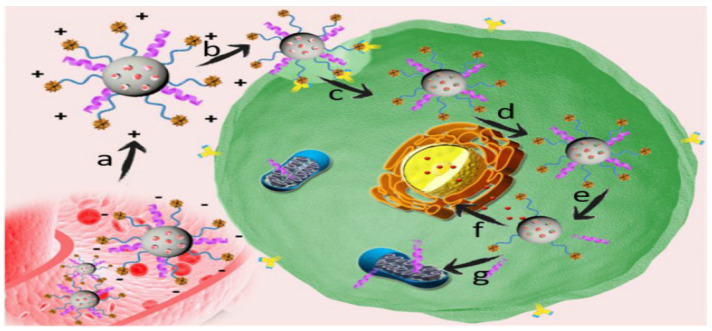
The delivery mechanisms of DOX@MSN-ss-DTPP&DTCPP nanoparticles: (**a**) recharging of DOX@MSN-ss-DTPP&DTCPP nanoparticles (NPs) in the microenvironment of acidic tumors; (**b**) Arg-Gly-Asp-Ser peptide (RGDS)-targeted aggregation of DOX@MSN-ss-DTPP&DTCPP NPs; (**c**) adsorption-reaction between DOX@ MSN-ss-TPP&TCPP NPs (positively charged) and negatively charged cell membrane; (**d**) cellular penetration of drug-loaded NPs to the tumor mediated by TCPP peptide; (**e**) removal of TPP and TCPP peptides from DOX-loaded NPs that caused by GSH; (**f**) release of DOX into the nucleus, leading to DNA damage; (**g**) GSH-triggered TPP peptide release into the mitochondria, producing a special mitochondrial disorder (Reprinted from [65], with permission from American Chemical Society).

The anti-cancer drug doxorubicin (DOX) has been loaded in the MSNs by physical-absorption. The NPs, prepared by MSN-ss-pyridyl NPs (100 mg), are dispersed in 10 mL of PBS buffer, where DOX, at 10 mg, is loaded under stirring, and the mixture interacts for 24 h at 25 °C in the dark. Then, a cancer-targeted fusion polypeptide, in combination with 2,3-dimethyl anhydride (DTCPP), and a therapy peptide with 2,3-dimethyl anhydride (DTPP), are attached to the surface of the MSNs via the disulfide bonds. The synthesized super pH-sensitive nanocarriers -DOX@MSN-ss-DTPP&DTCPP NPs are collected after centrifugation, washed with PBS buffer, and dried under vacuum [65]. The drug release from the DOX@MSN-ss-DTPP&DTCPP is shown to be influenced by the glutathione (GSH) and acidic state. The mechanism of absorption into the tumor cells and the ability of mitochondrial perturbation of DOX@MSN-ss-DTPP&DTCPP NPs in the location of the tumor is as depicted in Figure 9. The DOX@MSN-ss-DTPP&DTCPP has demonstrated higher cytotoxicity against HeLa cells with the IC_50_ of 1.4 µg/mL after 48 h treatment. The release of DOX and TPP peptide from DOX@MSN-ss-DTPP&DTCPP NPs can target the nucleus and mitochondria of HeLa cells at the same time, and exhibit stimulation of cell apoptosis [65]. 

**Figure 10:**
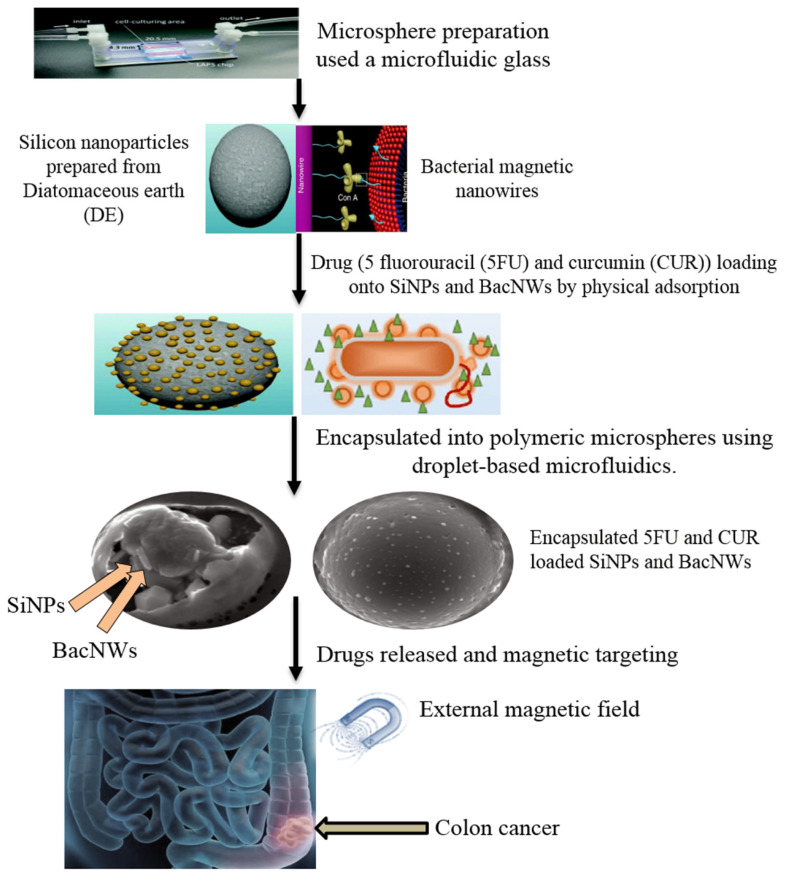
Anticancer drug (5-fluorouracil or curcumin) loaded silicon nanoparticles (SiNPs) and bacterial magnetic nanowires (BacNWs). The proposed application for combined therapy of colon cancer (Modified from [67]).

The use of silicon NPs (SiNPs) derived from diatoms has been evaluated for the delivery of DOX to the cancer cells [66]. The formation of nanoscale porous SiNPs is derived from the silica-DE by combining the magnesiothermic reduction with grinding and crushing by ultrasound. The prolonged and sustained release of DOX-loaded SiNPs is achieved for more than 30 days, with enhanced cytotoxicity of DOX-SiNPs on the MDA-MB-231-TXSA cells, as compared to the DOX alone. The release mechanism of DOX is based on the spread of the drug molecules from the nano-porous structure, and the decomposition of the silicon structure suggests the strong correlation between structural decomposition and drug release. The silicon-diatom-based carriers are developed from cheap and natural sources (DE) using an expandable transformation method, offering a promising alternative to produce synthetic silicon materials for the design of new nano-carriers [66]. Using micro-fluidics methods under an external magnetic field, as shown in Figure 10, combined SiNPs loaded with drugs and magnetic nanowires, and encased in a polymeric matrix sensitive to pH, have been synthesized to target SW480 colon cancer cells. The SiNPs and magnetic nano-wires (BacNWs) bearing Curcumin (CUR) or 5-Flourouracil (5FU) by using physical-adsorption, have resulted in strong synergistic activity of 5FU and CUR against SW480 cells. The hybrids reduce the 5FU dose, thus minimizing the side-effects, but with enhanced effectiveness due to the presence of CUR as an additional anticancer agent [67].

### 4.2. Diatoms-based Antibody

**Figure 11:**
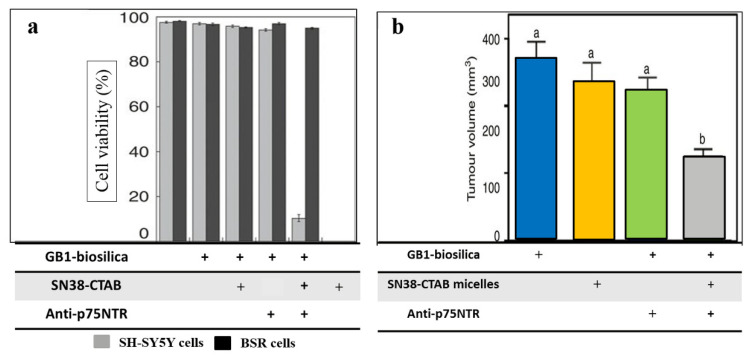
(**a**) The reduced cell viability of neuroblastoma cells in vitro after treatment with drug-loaded biosilica, and antibody-labelled biosilica; (**b**) The reduced neuroblastoma tumour growth *in vivo* after treatment with antibody-labelled diatoms biosilica loaded with SN38 (Modified from [61]).

A cancer-specific-antibody with drug-charged diatoms biosilica has been developed. *Thalassiosira pseudonana* is genetically designed to exhibit the IgG-binding field on the surface of the biosilica, allowing the binding of antibodies to target the cancer cells [61]. To target the neuroblastoma (SH-SY5Y) cancer cells, anti-p75NTR-loaded-GB1 biosilica frustules, which work faster against the antibody specific for p75 neurotrophin-receptor on the SH-SY5Y cells surface, is designed. The anti-p75NTR-loaded-GB1 biosilica reacts only with the SH-SY5Y cells, but not with the BSR fibroblast cells (derived from baby hamster kidney (BHK) cells) due to the lack of p75 neurotrophin-receptor. The drug-loaded IgG-biosilica is achieved by electrostatic adherence to the positively charged liposomes or micelles loaded with chemotherapy drugs such as camptothecin (CPT), or cetyltrimethylammonium bromide (CTAB), 7-ethyl-10-hydroxy-CPT (SN38). When this complex is incubated with the SH-SY5Y cells (having p75NTR antigen) and BSR cells, only the SH-SY5Y cells are significantly inhibited, but no effects on the BSR cells are observed (Figure 11a). For the in vivo study, the mice (BALB/c) that have SH-SY5Y cancer are treated with the injection of SN38-loaded and anti-p75NTR-loaded-biosilica. After five days, the tumor sizes in those treated mice are significantly reduced, as compared to the tumor-carrying mice treated only with the biosilica-microparticles lacking the antibody, or the drug, or both (Figure 11b). The major therapeutic effect is that of achieving tissue-specific treatment, as the biosilica-microparticles can only be discovered in the tumor tissue of mice treated with drug-loaded and antibody-loaded biosilica. No biosilica molecules are detected in the healthy or tumor tissues of mice treated with drug-bearing biosilica lacking the antibody. Therefore, the diatoms biosilica microparticles have proven to be effective for a targeted delivery of the chemotherapy drug in the in vivo mice model [68].

### 4.3. Diatoms-Based Vitamin B12

**Figure 12:**
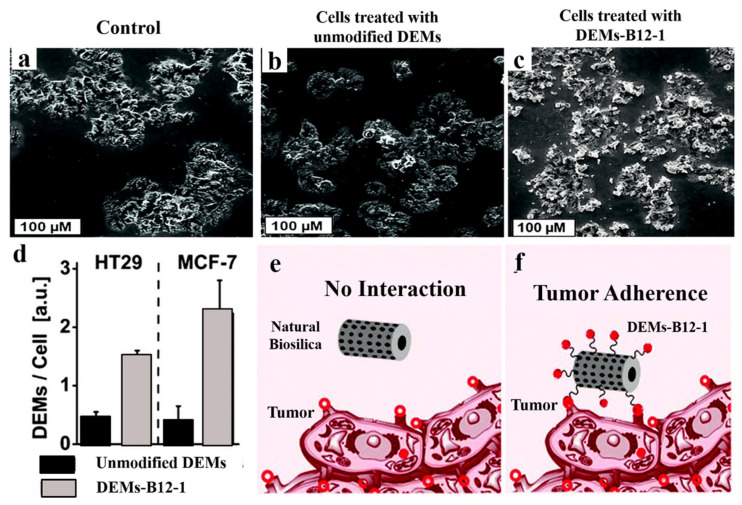
Scanning Electron Microscopic images of HT-29 cell line: (**a**) Control; (**b**) Treatment with 200 µg/mL of un-modified diatomaceous earth micro-particles (DEMs); (**c**) Treatment with 200 µg/mL of modified DEMs-B12-1; (**d**) The unmodified or modified DEMs treatment of HT29 and MCF-7 cells after 1 h exposure; (**e**,**f**) Schematic diagram of the DEMs per cell interaction (Modified from [10]).

Diatomaceous earth micro-particles (DEMs) isolated from diatoms can be used as a DDS for weakly water-soluble inorganic anti-cancer drugs. The DEMs surface modified with vitamin-B12 (tumor-targeting factor) and encapsulating the known anti-cancer drugs, including cisplatin, 5-fluorouracil (5-FU), and a tris-tetraethyl (2,2′-bipyridine)-4,4′-diamineruthenium(II) complex, have been evaluated for their cytotoxic effects [10]. The novel biomaterial developed is studied for enhanced adherence activity to the HT-29 and MCF-7 cancer cell lines. The interaction between the modified-B12 diatoms and the cancer cells is three times higher than the un-modified DEMs. This is related to the increased expression of transcobalamin II (TC (II)), and transcobalamin II receptors (TC (II)-R) in the target tissues. The different drugs loaded in the DEMs-B12-1 system show that the cisplatin and 5-FU are highly released. The ruthenium complex exhibits an unmatched release profile, preserved in the material for more than 5 days in the aqueous media, but easily released in the lipophilic media such as the cell membrane. The complex and 5-FU show higher anticancer activity as compared to the cisplatin (standard for inorganic anticancer drugs) against the HT29 cell line (Figure 12). This may indicate that DEMs-B12-1 is a promising delivery system for water-insoluble inorganic drugs to the cancer cells [10].

## 5. Conclusions and Future Work 

The marine diatoms are considered as an important source of novel anticancer agents with huge potential to be developed into diatoms-based anti-cancer drug delivery systems. Among the compounds derived from diatoms, monoacylglycerides, oxylipins, chrysolaminaran polysaccharide, adenosine, fucoxanthin, fatty alcohol ester, marennine, stigmasterol, and haslenes, have exhibited potent anticancer activities. The diatoms’ silica (hydrated-silicon dioxide), known as frustule, can be isolated and purified as a good source of cost-effective biosilica. With new synthesis and fabrication techniques developed on the silica shells of the diatoms with other biomaterials, and taking advantage of their unique chemical, structural, optical, and mechanical properties, diatomaceous earth particles offer better alternatives as anticancer drug carriers. The diatoms-based nanoparticles with antibody and vitamin B12 have shown to be tumor or cancer cell-specific, having longer drug retention and with reduced side-effects. The diatoms’ anticancer compounds and diatoms-based drug delivery system show promising avenues for further preclinical studies in the drug discovery programme and for clinical trials. In the future, these marine diatoms that are rich with cytotoxic compounds will be harnessed for the discovery of new pathways and lead compounds. The biosilica from diatoms can be further evaluated for their efficacy with cancer drugs to achieve targeted delivery and safe treatment. 

## Figures and Tables

**Table 1:** Summary of the bioactive compounds derived from different species of diatoms with anticancer activities on different cell lines.

Diatoms Species	Anticancer Compounds	Target Cells	IC_50_	Time	References
*Skeletonema marinoi*	Monoacylglycerides (MAGs)	Haematological cancer cell line (U-937)	5 µg/mL	24 h	[28]
		Colon cancer cell line (HCT-116)	5 µg/mL		
		MePR-2B normal cells	-		
	Polyunsaturated aldehydes (PUAs 2-trans,4-trans-decadienal (DD))	A549	Not clarified	48 h	[11]
		Colon adenocarcinoma metastatic ascites-deriving (COLO 205)	Not clarified		
		Normal lung/brunch epithelial (BEAS-2B)	-		
*Thalassiosira rotula* *Skeletonema costatum* *Pseudo-nitzschia delicatissima*	Unsaturated aldehydes	Caco-2 cells	11 ± 17 µg/mL	48 h	[35]
*Synedra acus*	Chrysolaminaran	Human colon cancer cells (HT-29)	54.5 µg/mL	72 h	[18]
		Colon cell line (DLD-1)	47.7 µg/mL		
*Phaeodactylum tricornutum*	Nonyl 8-acetoxy-6-methyloctanoate (NAMO, fatty alcohol ester)	Human promyelocytic leukemia (HL-60)	22.3 µg/mL	48 h	[3]
		Human lung carcinoma (A549)	50 µg/mL		
		Mouse melanoma (B16F10)	-		
	Monogalactosyl diacylglycerols	Wild-type W2 Wild-type D3	52 µM 64 µM	48 h	[36]
	Fucoxanthin	Caco-2 (derived from a human colon adenocarcinoma), Hep G2, and HeLa (derived from cervical cancer cells)	Not clarified	48 h	[37]
*Navicula incerta*	Stigmasterol (phytosterol)	Liver hepatocellular carcinoma (HepG2)	8.25 µg/mL	24 h	[38]
*Haslea ostreria*	hasla-6(17),9,13,23- tetraene	Human lung cancer (NSCLC-N6)	3.8 µg/mL	72 h	[39]
	Marennine	Skin cancer (M96), lung cancer (NSCLC-N6), and kidney cancer (E39)	30.2, 34.2, and 57.8 µg/mL		
*Cocconeis scutellum*	Fraction 3 (eicosapentaenoic acid (EPA), diethyl ether extract)	Breast carcinoma (BT20) Human normal lymphocytes	Not clarified -	24 h	[40]
*Chaetoceros calcitrans*	EtOH extract	MCF-7	3 µg/mL	24 h	[41]
	AcOEt extract	Breast adenocarcinoma (MDA-MB-231)	60 µg/mL	72 h	[42]
